# Elevated Micro- and Nanoplastics Detected in Preterm Human Placentae

**DOI:** 10.21203/rs.3.rs-5903715/v1

**Published:** 2025-02-03

**Authors:** Michael Jochum, Marcus Garcia, Alexandra Hammerquist, Jacquelyne Howell, Myla Stanford, Rui Liu, Marian Olewine, Eliane El Hayek, Emily Phan, Lori Showalter, Cynthia Shope, Melissa Suter, Matthew Campen, Kjersti Aagaard, Enrico Barrozo

**Affiliations:** Department of Obstetrics and Gynecology, Baylor College of Medicine and Texas Children’s Hospital; University of New Mexico; Department of Obstetrics and Gynecology, Baylor College of Medicine and Texas Children’s Hospital; Department of Obstetrics and Gynecology, Baylor College of Medicine and Texas Children’s Hospital; Department of Obstetrics and Gynecology, Baylor College of Medicine and Texas Children’s Hospital; University of New Mexico; University of New Mexico; University of New Mexico; Department of Pharmaceutical Sciences, University of New Mexico; Department of Obstetrics and Gynecology, Baylor College of Medicine and Texas Children’s Hospital; Department of Obstetrics and Gynecology, Baylor College of Medicine and Texas Children’s Hospital; Baylor College of Medicine, Department of Obstetrics & Gynecology; University of New Mexico; Oregon National Primate Research Center & HCA Healthcare and HCA Healthcare Research Institute & Boston Children’s Hospital, Harvard Medical School; Baylor College of Medicine and Texas Children’s Hospital

**Keywords:** microplastic, preterm birth, obstetrics outcomes, developmental toxicology, environmental pollutants

## Abstract

Recent analytical advancements have uncovered increasing micro- and nanoplastics (MNPs) in environmental, dietary, and biological domains, raising concerns about their health impacts. Preterm birth (PTB), a leading cause of maternal and neonatal morbidity and mortality, may be influenced by MNP exposure, yet this relationship remains unexplored. This study quantified 12 MNP polymers in placentae from term (n=87) and preterm (n=71) deliveries using pyrolysis-gas chromatography/mass spectrometry (Py-GC/MS). Cumulative MNP concentrations were 28% higher in PTB placentae (mean ±SD: 224.7 ± 180.7 μg/g vs. 175.5 ± 137.9 μg/g; p=0.038). Polyvinyl chloride (PVC), polyethylene terephthalate (PET), polyurethane (PU), and polycarbonate (PC) were significantly elevated in PTB, and PET, PU, and PC inversely correlated with gestational age and birth weight. Logistic regression identified PVC and PC as independent predictors of PTB. These findings suggest total and specific MNPs are associated with PTB, providing actionable insights and emphasizing the importance of minimizing exposure during pregnancy.

## Introduction

Micro- and nanoplastics (MNPs) have emerged as significant environmental contaminants due to their widespread use and persistence in the environment. Humans are estimated to ingest and absorb growing quantities of MNPs through food, water, and air.^1,2^ This pervasive exposure raises concerns regarding the potential health impacts of MNPs on vulnerable populations, such as pregnant women and developing fetuses. While MNPs have been detected in human tissues and fluids,^3–14^ their long-term effects, especially during pregnancy, remain poorly understood.

Preterm birth (PTB), a leading cause of maternal and early-life mortality and morbidity^15^, is a major global health challenge. Despite multiple decades of research, there are no effective interventions, and the spontaneous PTB rate remains effectively unchanged, affecting approximately 10% of pregnancies worldwide and incurring an estimated $25 billion in healthcare costs in the United States.^16,17^ Despite decades of research, most cases of spontaneous (non-medically indicated) PTB remain idiopathic, with growing evidence implicating both vascular and inflammatory processes as potential drivers.^18–21^ Given their putative causal role in modulating atherosclerotic disease,^22^ a potential role for MNPs exposure in driving aberrant placental pathophysiology and triggering PTB remains a critical and understudied area of maternal-fetal health research.

Environmental toxicants such as air pollution, heavy metals (e.g., lead and cadmium), endocrine-disrupting chemicals (e.g., phthalates and bisphenol A), and polycyclic aromatic hydrocarbons (PAHs) have been implicated in PTB.^23–31^ Many of these toxicants can accumulate and cross the placenta, disrupting placental function, inducing oxidative stress, and contributing to adverse pregnancy outcomes. Despite these insights, MNPs, which share many characteristics with these toxicants, have yet to be thoroughly investigated for their role in PTB.

In 2021, MNPs were first identified in human placentae, providing critical evidence that they can reach the maternal-fetal barrier.^12^ Subsequent studies have shown that MNPs are ubiquitous in placental tissues at varying concentrations^11,32–41^ and have been detected in amniotic fluid, cord blood, and meconium^11,34,35,37,41,42^. However, there currently are limited studies that have explored the relationship between MNP exposure and health outcomes. Recently, significantly higher concentrations of MNPs were reported in placentae from fetal growth-restricted pregnancies compared to controls, with inverse associations noted between MNP levels and birth weight.^32^ Emerging evidence also implicates MNPs in recurrent pregnancy loss, with a study using pyrolysis gas chromatography-mass spectrometry (Py-GC/MS) reporting significantly higher polystyrene (PS) concentrations in placentae from miscarriage cases compared to controls (odds ratio of 34, 95% CI: 3.61–320).^40^

Despite this growing body of preclinical evidence, significant gaps remain in understanding if and how MNPs contribute to adverse gestational outcomes such as PTB. In this study, we hypothesized that placental MNP concentrations and polymer profiles differ between term and PTBs. Using Py-GC/MS, we quantified 12 types of MNPs in *N* = 158 human placentae, focusing on their associations with gestational age at delivery, maternal characteristics, and perinatal outcomes. Our findings reveal novel insights into the accelerated bioaccumulation of MNPs in preterm placentae, their correlations with gestational age, and their potential associations with maternal conditions known to exacerbate pathways critical to labor initiation.

## Results

### Clinical and demographic characteristics of the cohort.

The study comprised 158 subjects stratified by gestational age at delivery: Term (≥ 37 weeks; *n* = 87) and Preterm (< 37 weeks; *n* = 71). Nesting of the cohort and study design assured an equal distribution of preterm and term deliveries, equal representation across ethnicity and race (non-Hispanic White, Hispanic White, non-Hispanic Black, and non-Hispanic Asian), an equal fetal sex ratio, cesarean delivery, and live birth. Table 1 summarizes the clinical and demographic characteristics of the cohort (relevant clinical metadata available in Table S1a). Maternal race and ethnicity differed significantly between groups (*p* < 0.001), with a higher proportion of Hispanic White participants in the PTB group (70.8%) compared to the Term group (28.7%) and a lower proportion of non-Hispanic Black participants in the PTB group (12.5%) compared to the Term group (20.7%; *p* = 0.019). As anticipated, hypertension was more prevalent in the PTB group (28.2%) than in the Term group (8.0%; *p* = 0.002). Similarly, the Social Deprivation Index (SDI) was higher in the PTB group (79.62 ± 23.29) compared to the Term group (70.32 ± 28.72; *p* = 0.04). Prior PTBs were more frequent in the PTB group (23.9%) than in the Term group (9.2%; *p* = 0.021). Similarly and as anticipated, the type of labor also differed significantly (*p < 0.001*), with more spontaneous labor in the PTB group (21.1%) than in the Term group (8.0%; *p = 0.0*33), higher rates of preeclampsia (35.2% vs. 4.6%; *p* < 0.001) and PPROM (14.1% vs. 0%; *p* = 0.001) in the PTB group. Medical co-morbidities accompanying cesarean delivery were significantly different (*p* < 0.001), while other medical indications were more common in the Term group (10.3% vs. 1.4%; *p* = 0.049). As expected, gestational age at delivery (34.72 ± 2.11 weeks vs. 39.32 ± 1.39 weeks; *p* < 0.001) and birth weight (2499.87 ± 674.09 g vs. 3406.91 ± 470.70 g; *p < 0.001*) were significantly different between preterm and term deliveries, respectively. APGAR scores at 1 minute were lower in PTB deliveries (7.34 ± 1.93) compared to Term deliveries (7.89 ± 1.63; *p* = 0.04), which is not considered a clinically meaningful difference, despite the statistical difference. Potential confounders of PTB, such as prior PTBs, preeclampsia, and racial health disparities, were controlled for in subsequent univariate and multivariate analyses, ensuring robust comparisons.

### Despite shorter gestations, PTB placentae have higher concentrations of MNPs than those delivered at term.

**Figure 1:**
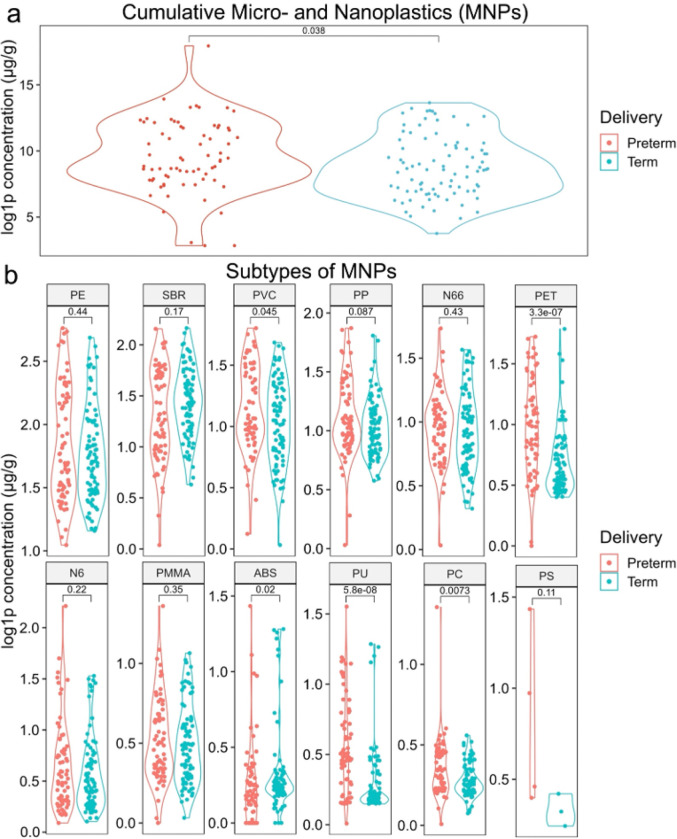
Despite shorter gestations, preterm placentae have higher concentrations of MNPs than those delivered at term. **a-b,** Violin plots comparing (**a**) the cumulative concentration of MNPs or (**b**) subtypes of MNPs. Individual points are jittered on each violin plot, with preterm denoted in red and term denoted in blue. Statistical comparisons were made using Wilcoxon tests with a significance of *p*<0.05.

Placental specimens were subjected to Py-GC/MS to quantify twelve types of MNPs: polyethylene (PE), styrene-butadiene rubber (SBR), polyvinyl chloride (PVC), polypropylene (PP), nylon 66 (N66), polyethylene terephthalate (PET), nylon 6 (N6), polymethyl methacrylate (PMMA), acrylonitrile butadiene styrene (ABS), polyurethane (PU), polycarbonate (PC), and PS. Samples were run in duplicate and quantified relative to standards (Py-GC/MS parameters, settings, and standards available in Table S1b). The cumulative concentrations of MNPs were log1p transformed^43^ and assessed comparing preterm versus term delivery status, revealing MNP concentrations in PTB placentae were 28% higher (Fig. 1a, mean ± SD: 224.7 ± 180.7 μg/g vs. 175.5 ± 137.9 μg/g; *p* = 0.038, Wilcoxon test). Individual MNP concentrations were analyzed in Table 2 and Fig. 1b, identifying significantly higher MNP types in PTB placentae, including PVC (*p* = 0.045; 17% higher), PET (*p* < 0.001; 113% higher), PU (*p* < 0.001; 157% higher), and PC (*p* = 0.007; 46% higher). Only ABS was higher in term placentae (*p* = 0.02; 42% higher).

### Placental MNP concentrations are associated with maternal comorbid conditions and environmental factors.

**Figure 2:**
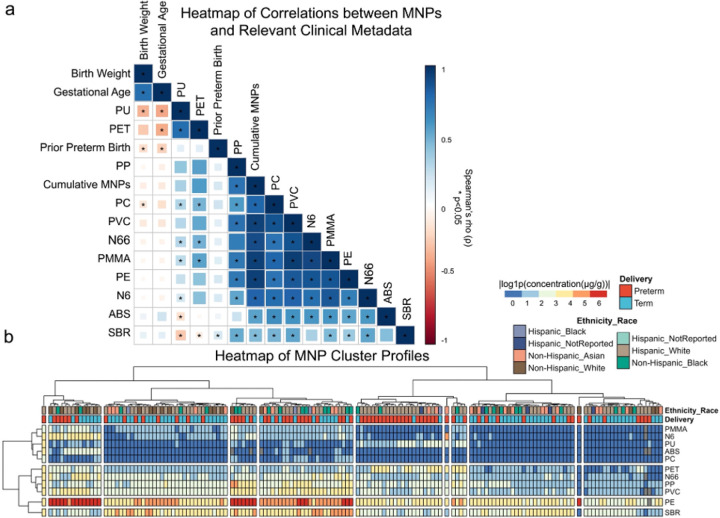
Specific MNPs correlate with birth weight and gestational age, while MNP concentration profiles cluster by preterm delivery status. **a,** Correlation matrix of cumulative and specific MNP concentrations with birth weight, gestational age, and prior PTB. Spearman’s correlation analysis with Benjamini-Hochberg corrections was used to determine significance, denoted by * for *q*<0.05.**b,** Heatmap of MNP concentrations stratified by delivery status. Data were log-transformed (log1p) to stabilize variability and approximate a normal distribution. Rows represent MNP measurements, and columns correspond to individual samples. Clustering of rows and columns was performed using Euclidean distance and Ward’s D2 method to highlight patterns within and across groups.

Spearman’s correlation analyses were performed to examine associations between placental MNP concentrations and clinical metadata, including maternal comorbidities (Fig. 2a; Table S1c). Cumulative MNPs showed significant positive correlations with PC, PVC, N6, PMMA, PE, N66, ABS, and SBR (*ρ* = 0.44–0.93, *p* < 0.001). Additionally, significant inverse correlations were identified between birth weight with placental PU (*ρ*=−0.35, *p* = 0.001) and PC (*ρ*=−0.14, *p* = 0.048) concentrations.

Wilcoxon tests revealed elevated PET (p = 0.031) and PU (*p* = 0.053) in placentae from subjects with preeclampsia (n = 29; Fig S1a). Placentae from individuals with a smoking history (n = 11; Fig S1b) showed increased cumulative MNPs (*p* = 0.0065), PE (*p* = 0.0029), and PP (*p* = 0.058). In contrast, placentae from participants with gestational diabetes exhibited lower levels of cumulative MNPs (*p* = 0.057), N6 (*p* = 0.0051), PC (*p* = 0.026), PE (*p* = 0.028), PVC (*p* = 0.032), PMMA (*p* = 0.046), and ABS (*p* = 0.047). These findings demonstrate that placental MNP concentrations are significantly associated with key maternal comorbidities and environmental factors, highlighting the unlikely probability of their being mere contaminants and emphasizing the need for further investigation into their robust independent association with maternal health.

### Clustering analysis reveals predictability of patterns in MNP profiles and delivery classifications.

To explore patterns in MNP distributions, concentration data were categorized by delivery classifications (term or preterm) and ethnicity and race (Fig. 2b). Clustering analysis, performed using Euclidean distance and Ward’s D2 method, revealed distinct patterns within and across groups. This approach highlighted co-occurrence trends among MNPs and their association with demographic and clinical variables, providing a basis for further exploration of their roles in pregnancy outcomes. Subjects were grouped into 11 clusters, while MNPs formed 4 distinct clusters. PE and SBR separated into their own clusters, suggesting unique accumulation or exposure pathways. PMMA, N6, PU, ABS, and PC clustered together, indicating potential shared sources or biological interactions, while PET, N6, PP, and PVC formed another distinct group. These clustering results suggest that specific and groups of MNPs may have shared mechanisms of accumulation or exposure and provide hypotheses for investigating their roles in pregnancy outcomes.

### Gestational age at delivery correlates with specific MNP concentrations.

**Figure 3:**
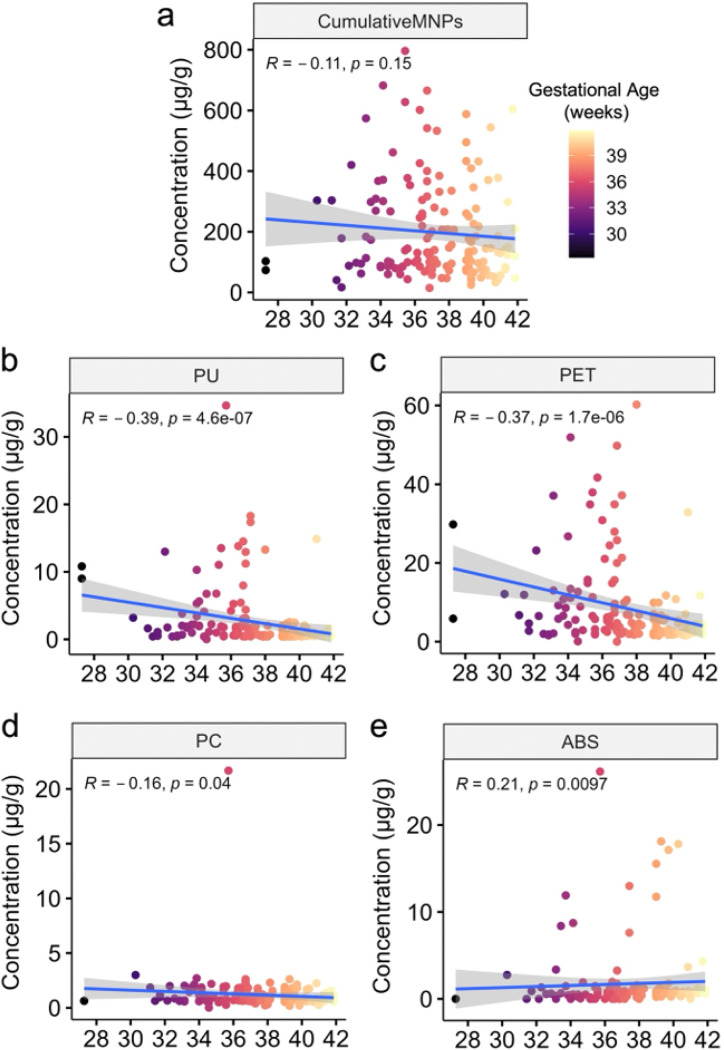
Gestational age at delivery negatively correlates with PU, PET, and PC levels, positively correlates with ABS levels, and shows no significant correlation with cumulative MNPs. **a-e,** Scatterplots of gestational age at delivery and: (**a**) cumulative MNPs, (**b**) PU, (**c**), PET, (**d**) PC, and (**e**) ABS concentrations. Spearman’s correlation ρ and significance defined as *p*<0.05 visualized with 95% confidence intervals. Individual subject data are depicted with dots colored by gestational age at delivery.

For the comparison of cumulative MNPs, we applied a log transformation (log1p) to stabilize variance and approximate a more normal distribution. Gestational age at delivery showed significant differential correlations with individual MNP levels but no significant correlation with cumulative MNPs, suggesting that potential laboratory environmental contamination is unlikely to contribute to our findings. Specifically, cumulative MNPs did not significantly correlate with gestational age (Fig. 3a; *ρ*=−0.11, *p* = 0.15). However, significant inverse correlations were observed for PU (*ρ*=−0.39, *p* < 0.001), PET (*ρ*=−0.37, *p* < 0.001), and PC (*ρ*=−0.16, p = 0.04) (Fig. 3c–e). In contrast, ABS was the only MNP to positively correlate with gestational age at delivery (Fig. 3f; *ρ* = 0.21, *p* = 0.01). These findings suggest that while cumulative MNPs do not correlate with gestational age, specific MNPs may influence or reflect differences in pregnancy duration.

### Logistic regression analysis identifies associations between MNP concentrations and PTB.

**Figure 4:**
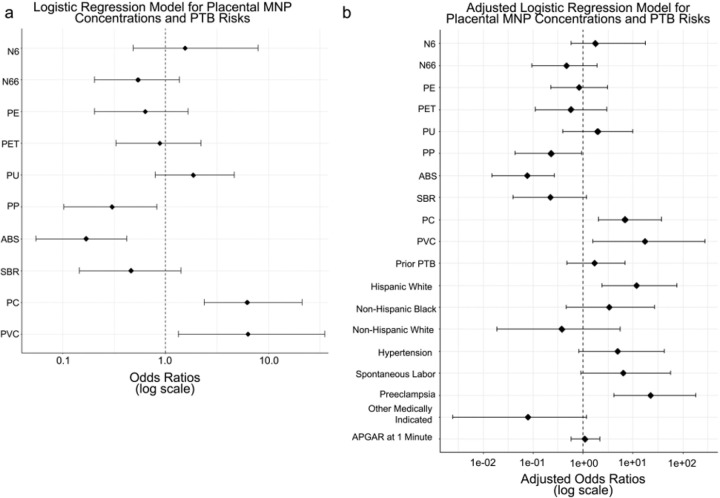
Logistic regression analysis identifies associations between MNP concentrations and PTB. **a-b,** Forest plots showing the unadjusted (**a**) or adjusted (**b**) odds ratios (OR) and 95% confidence intervals (CIs) for predictors of preterm birth based on placental MNP concentrations. Odds ratios are plotted on a logarithmic scale, with the dashed vertical line representing the null effect (OR = 1). Each point denotes the OR, while horizontal lines indicate the 95% CI.

To comprehensively evaluate the PTB risks associated with MNPs, an unadjusted model was compared with an adjusted model accounting for collinearity in MNP exposure (Fig. 2b) and potentially confounding PTB comorbidities (Table 1). In the unadjusted model, SBR, PVC, ABS, PP, and PC were significant predictors (Fig. 4a; *p* < 0.05). Model performance comparison demonstrated the adjusted model’s superiority, with a markedly lower AIC (124.31) and residual deviance (82.31) compared to the unadjusted model (Akaike Information Criterion (AIC) = 175.11, residual deviance = 153.11). Incorporating clinical metadata in the adjusted model, significant predictors included PVC, ABS, PP, PC, Hispanic White ethnicity, spontaneous labor, and preeclampsia (Fig. 4b; *p* < 0.05). In both models, ABS and SBR had negative β values and odds ratios < 0.23, consistent with their inverse correlations with gestational age at delivery (Figs. 2–3). Conversely, PVC and PC had positive β values and odds ratios > 6.9, aligning with their positive correlations with PTB risk (Figs. 2–3). These results underscore the critical importance of integrating clinical metadata to refine risk models and highlight specific MNPs, particularly PVC and PC, as significant and independent predictors of PTB risks, corroborating findings from univariate analyses.

## Discussion

This study demonstrates that placental MNP concentrations are significantly elevated in placentae from preterm deliveries. Cumulative MNP levels were 28% higher in preterm placentae, despite there being a mean of 4.6 weeks less time for accumulation compared to term placentae (term gestation is 37 weeks, representing > 12% less time for bioaccumulation, on average). Specific MNPs such as PVC, PET, PU, and PC showed significant elevations in PTB, while ABS was higher in term placentae. Thus, the specificity and inverse accumulation by gestational time make environmental or laboratory contamination highly unlikely. MNP concentrations correlated with maternal comorbid conditions which are associated with PTB, including elevated PET and PU in preeclamptic placentae, increased cumulative MNPs, PE, and PP in cases with a smoking history, and reduced cumulative MNPs and multiple individual MNPs in gestational diabetes. Clustering analyses revealed distinct patterns of MNP accumulation by delivery classification, highlighting potential shared pathways of exposure or biological interactions. Together, these findings provide initial and robust evidence that the accumulation of specific MNPs in the placenta is associated with adverse pregnancy outcomes, including PTB, warranting further causative investigations.

This study aligns with prior studies that reported elevated MNPs in adverse pregnancy outcomes, such as intrauterine fetal growth restriction and recurrent pregnancy loss^32,40^ and expands on them by showing significantly higher cumulative and specific MNP concentrations in preterm placentae. MNPs such as PET and PU demonstrated strong negative correlations with gestational age, corroborating studies linking MNP exposure to oxidative stress, placental dysfunction, and aberrant vascular and immune pathophysiology.^5,44–47^ The association between MNPs and maternal comorbid conditions, such as preeclampsia and smoking, suggests a complex interaction between environmental exposures and maternal health conditions.

Few studies have explored the associations between MNPs and human placental histopathology. One study identified ultrastructural alterations in placentae associated with MNPs utilizing variable pressure scanning electron microscopy and transmission electron microscopy.^33^ Another study using Py-GC/MS reported significantly higher PS concentrations in placentae from recurrent pregnancy loss cases compared to controls and detected increased apoptosis in recurrent pregnancy loss placentae using a TUNEL assay.^40^ In trophoblast cell cultures, PS nanoparticles reduced Bcl-2 and mitochondrial membrane potential while increasing reactive oxygen species and cleaved caspase-2 and -3.^40^ In a murine model of recurrent pregnancy loss^40^, daily exposure to 50 or 100 mg/kg of PS for 14 days increased fetal demise, while 25 mg/kg/day had no effect. Supplementing Bcl-2 protected against PS-induced trophoblast apoptosis and fetal demise in the mouse model.^40^

While MNPs are known to disrupt normal pathophysiology, their influence on placental pathophysiology remains uncharacterized. Rodent models have demonstrated the potential for MNPs to cause pathogenesis during pregnancy. Exposure to MNPs resulted in a range of phenotypes, including metabolic disorders, trophoblast apoptosis, placental dysfunction, intrauterine fetal growth restriction, and fetal demise (stillbirth).^40,48–51^ PS MNPs have been shown to cross the placenta, induce oxidative stress, and disrupt maternal-fetal immune and vascular physiology.^48,52^ For example, Hu et al. (2021) demonstrated that PS exposure induced fetal demise, reduced decidual NK cells, increased helper T cells, shifted placental macrophage polarization toward anti-inflammatory M2, and led to immunosuppressive cytokine profiles.^48^ Notably, some effects of MNP exposure, including transgenerational impacts on metabolic health, extend beyond the F1 generation.^51^ Studies using human clinical data and longitudinally collected specimens are essential to uncover how MNPs influence pregnancy outcomes, contribute to the developmental origins of health and disease, and validate animal model findings in the human context.

The observed associations between MNPs and PTB, as well as conditions like preeclampsia and smoking, have important clinical implications. Screening for MNPs in placental or maternal blood samples could help identify pregnancies at risk for adverse outcomes. The association between smoking history and elevated MNPs highlights a compounded risk of behavioral and environmental exposures, reinforcing the importance of targeted interventions and public health campaigns aimed at reducing exposure during pregnancy. Furthermore, the inverse correlation between gestational age and specific MNPs, such as PU and PET, suggests that reducing environmental exposure to these plastics may mitigate risks for preterm delivery. MNPs have previously been associated with gestational age, though it is unclear whether PTB cases were included in these studies. An inverse correlation has previously been reported between placental MNPs detected by Raman microspectroscopy (*n* = 43 placentae, 13 FGR; 2–38 particles/placenta) and birth weight, length, head circumference, and 1-minute APGAR scores.^32^ A significant negative association between MNPs in amniotic fluid (*n* = 40 subjects) and gestational age and birth weight was observed by LD-IR.^42^ These findings call for the development of guidelines to limit environmental plastic exposure in vulnerable populations, particularly pregnant women.

The use of Py-GC/MS for MNP analysis offers a standardized and highly sensitive method for detecting and quantifying MNPs, addressing limitations of earlier techniques and ensuring reliable results. Consistently, MNP concentrations detected by Py-GC/MS in biological specimens from human placentae, brains, kidneys, testes, and livers are at significantly higher concentrations than non-existent or trace levels in operator, environment, and technical blank control samples.^4,9,14,39,53,54^ Additionally, placental MNP accumulation has demonstrably been shown to increase over time^38^, suggesting systematic technical contamination could not be the source of the MNPs detected in the preterm placentae. Lastly, Py-GC/MS is limited in not providing information on the numbers of plastic particles, but the cumulative assessment of all nanoscale polymers is an important advantage, given recent evidence that submicron particles may reflect the major mass balance of MNPs in biological tissues.^4,9,14,39,53–55^

The strengths of this study include its robust nested cohort design, rigorous methodology, and large sample size. However, while the cohort size was sufficient to detect significant associations, it may be underpowered to identify relationships with MNPs at low concentrations or smaller effect sizes. The prospective observational design limits causal inference, and unmeasured confounders, such as dietary habits or regional exposures, may influence findings. Placental tissue, while a practical proxy, may not fully capture dynamic exposure pathways or the timing of MNP accumulation, and the cross-sectional nature of this nested cohort precludes assessment of temporal variability. Differences between univariate and multivariate analyses highlight potential co-linearity among the polymers, which is consistent with recent findings and may reflect environmental MNP concentrations.^4,9,14,39,53,54^ Multivariate models demonstrated larger effect sizes for MNPs such as PVC, N6, and PC, suggesting robust associations after adjustment but also raising questions about shared exposure pathways with clinical factors like preeclampsia and smoking. These findings emphasize the complexity of MNP impacts and the need for statistical approaches to disentangle overlapping effects. While 12 MNP polymer types were assessed, additional environmental exposures could be measured. Additionally, while the cohort’s diversity is a strength, findings may not generalize to other populations with differing exposures or healthcare access. Future research should incorporate larger, longitudinal cohorts and complementary analytical techniques to validate these findings and address residual confounding.

In conclusion, this study provides compelling evidence of elevated placental MNP concentrations in PTBs, with specific MNPs showing strong associations with maternal comorbid conditions and delivery outcomes. By identifying correlations between MNPs and perinatal outcomes, this study highlights the importance of environmental toxicants in shaping maternal-fetal health. The findings emphasize the need for mechanistic research to elucidate the biological pathways by which specific MNPs influence pregnancy and reproductive outcomes. Clinically, this study supports the development of public health strategies aimed at minimizing environmental exposures in pregnancy to reduce the burden of PTB and associated complications.

## Methods

### Inclusion and ethics statement.

The authors declare no competing financial or non-financial interests. All authors agree with the manuscript contents, the author list, the author order, and the respective contributions. The composition and procedures of this study’s investigators and subjects align with the Baylor College of Medicine Mission, Vision, and Values, including the Nondiscrimination Policy prohibiting discrimination on the basis of race, ethnicity, age, religion, disability, gender, gender identity, or expression, sexual orientation, nationality, or veteran status.

### Statement of compliance.

The human placentae, collected from 2011 to 2019, were obtained with informed consent and sourced from the Baylor College of Medicine Perinatal Biobank (PeriBank)^24,39,56–76^, governed by protocol H-26364. The subsequent analysis of MNPs in these coded banked samples was also sanctioned by the Baylor College of Medicine IRB (Protocols H-30688, H55700, and H-55735) as participants in PeriBank granted unrestricted permission for future research use. The University of New Mexico Human Research Protections Office also reviewed and approved the study as exempt.

### Subjects and sample collection.

The study population consisted of 159 subjects prospectively enrolled with their placentae and tissue banked for future research. The current study cohort was nested and stratified by gestational age at delivery: Term (≥ 37 weeks; *n* = 87) and Preterm (< 37 weeks; *n* = 72) (Table 1; relevant clinical metadata, including labor characteristics, are detailed in Table S1a). Subjects were recruited by PeriBank study personnel at admission to labor and delivery. After obtaining consent, placental samples were collected and rigorously processed, and clinical metadata—including up to 4,700 variables—was extracted from electronic medical records, prenatal records, and directed subject interviews. Data extraction was conducted by trained nurses and research staff, with routine audits by a maternal-fetal medicine physician-scientist (K.M.A.) to ensure quality. Inclusion criteria included 50% preterm and 50% term deliveries, equitable distribution by ethnicity and race (non-Hispanic White, Hispanic White, non-Hispanic Black, and non-Hispanic Asian), an equal fetal sex ratio, cesarean delivery, and live birth. Exclusion criteria included multiparous pregnancies, congenital malformations, fetal anomalies, and cancer. Social deprivation indices (SDI) from 2011–2018 ACS data were incorporated using Zip Codes at the time of delivery.

To ensure sufficient statistical power for detecting significant associations between MNP concentrations and PTB, we conducted a power analysis based on previously published data (*n* = 3 preterm, *n* = 57 term).⁴⁰ Significant differences in the concentrations of three MNPs—PVC, PU, and N6—were identified, with effect sizes (Cohen’s *d*) of 1.44 for PVC, −1.19 for PU, and 1.28 for N6. Using these effect sizes, we calculated the sample sizes required to achieve 80% power at an alpha of 0.05: 9 subjects for PVC, 13 for PU, and 11 for N6. To ensure robustness, we aimed to enroll the maximum required sample size across all three MNPs (26 subjects: 13 term and 13 preterm). The current study exceeds this target with 159 subjects, providing ample power for detecting meaningful associations between MNP concentrations and PTB.

As previously described^39^, samples were circumferentially excised 4 cm from the cord insertion site, avoiding maternal decidua to minimize contamination. Dissected sections were placed in sterile polyurethane tubes and immediately flash-frozen at −80°C. Specimen handling adhered to standardized sterile techniques to ensure quality for specimen banking in PeriBank. All specimens used in the current study were primary aliquots stored at −80°C since collection and had not undergone freeze-thawing.

### Placenta digestion for MNP purification.

Placenta samples, each approximately 0.38g, were digested using a 10% potassium hydroxide (KOH) solution in a 3:1 volume ratio as described previously.^39^ The digestion was conducted in glass vials, incubated at 40°C with continuous agitation for 72 hours. Post-digestion, the supernatant was transferred to ultracentrifuge tubes, to which 200μl of 100% ethanol was added. Ultracentrifugation at 100,000g for 4 hours separated MNP pellets from the supernatant. These pellets were washed thrice with 100% ethanol and air-dried for 24 hours at room temperature. The dried samples were stored in glass vials for subsequent Py-GC/MS quantitative analysis.

### Pyrolysis Gas Chromatography/Mass Spectrometry (Py-GC/MS) quantification.

Py-GC/MS analyses were conducted using an Agilent 6890 GC/5975 MS system with an EGA/PY-3030D Pyrolysis unit (Frontier Labs, Koriyama, Japan) and a UAMP Column kit for MNP analysis. Placental samples were weighed using an EPE26 Precision Balance (Mettler Toledo), placed in stainless-steel Eco-cup SF sample cups (Frontier Labs, Koriyama, Japan), and subjected to pyrolysis. The pyrolysis parameters, including temperature, hold time, and helium carrier gas flow rate, were controlled via F-Search MPs software v2.1 (Frontier Labs, Koriyama, Japan). Samples were heated to 600°C, with volatile products from polymer degradation captured and analyzed. The total runtime per sample was approximately 45 minutes. Data were normalized to the placental tissue weights. The GC/MS analysis utilized a UAMP column for separating pyrolysis products, targeting twelve specific polymers. MNP quantification was achieved through F-Search MPs 2.1 software (Frontier Labs, Koriyama, Japan). Identification and quantification were based on mass spectra and retention times, using a calibration curve constructed from calcium carbonate MNP polymer standards (Frontier Labs, Koriyama, Japan) at varying weights (0.1mg to 4mg). This calibration facilitated the analysis and quantification of MNPs in biospecimen samples using the software, ensuring precise measurements for each tissue sample examined. Subject MNP concentrations are available in Table S1a. Extracted Ion Chromatogram Spectrum of the 12 MNP polymers of interest and detailed Py-GC/MS parameters are available in Table S1b.

### Statistical analyses.

Statistical analyses were performed in R (v4.3.1). Descriptive statistics (mean, standard deviation, etc.) of raw MNP distributions were calculated (Table 2). Group and pairwise comparisons were evaluated using Wilcoxon or Kruskal-Wallis tests with Benjamini-Hochberg corrections for multiple comparisons. Categorical variables were assessed using Chi-Square tests. Continuous variables were scaled, log1p normalized, and analyzed using Spearman’s correlation coefficient with Benjamini-Hochberg corrections, with significance set at an adjusted *p*-value (*q*) < 0.05 (Table S1c).

Multivariable logistic regression was conducted using the glm() function in R with a binomial family to model the binary outcome. Predictor variables included MNP concentrations (unadjusted model) or adjusted by excluding variables with > 85% collinearity and accounting for maternal and fetal characteristics such as preeclampsia, hypertension, labor type, and APGAR scores (Table 1; Table S1d). Multicollinearity was assessed using Variance Inflation Factor (VIF), with a threshold of > 10 indicating potential collinearity. Adjusted odds ratios (aORs) and 95% confidence intervals (CIs) were derived by exponentiating the model coefficients and their confidence intervals. Statistical significance was defined as *p* < 0.05. Model fit was assessed using the Akaike Information Criterion (AIC) and residual deviance.

## Figures and Tables

**Table 1:** Clinical and Demographic Characteristics of the Study Cohort by Delivery Term Classification.

Variable	Overall Cohort (*N* = 158)	Preterm Group (*n* = 71)	Term Group (*n* = 87)	*p*-value
Maternal Age, years	31.71 ± 6.44	32.14 ± 6.37	31.35 ± 6.51	0.441
Ethnicity/Race:				**<0.001**
Hispanic White	76 (47.8%)	51 (70.8%)	25 (28.7%)	**<0.001**
Non-Hispanic Black	27 (17.0%)	9 (12.5%)	18 (20.7%)	**0.019**
Non-Hispanic Asian	24 (15.1%)	5 (6.9%)	19 (21.8%)	0.263
Non-Hispanic White	23 (14.5%)	5 (6.9%)	18 (20.7%)	**0.028**
Hispanic Black	4 (2.5%)	1 (1.4%)	3 (3.4%)	1.000
Hispanic/Race Not Reported	5 (3.1%)	1 (1.4%)	4 (4.6%)	1.000
Hypertension	27 (17.1%)	20 (28.2%)	7 (8.0%)	**0.002**
Social Deprivation Index (SDI)	74.45 ± 26.77	79.62 ± 23.29	70.32 ± 28.72	**0.040**
Maternal Smoking, never	147 (93.0%)	65 (91.5%)	82 (94.3%)	0.726
Gestational Diabetes, yes	31 (19.6%)	18 (25.4%)	13 (14.9%)	0.151
Gravida	2.95 ± 1.80	2.86 ± 1.56	3.02 ± 1.98	0.165
Prior PTB, yes	25 (15.8%)	17 (23.9%)	8 (9.19%)	**0.021**
Preeclampsia	29 (18.4%)	25 (35.2%)	4 (4.6%)	**<0.001**
PPROM	10 (6.3%)	10 (14.1%)	0 (0.0%)	**0.001**
Type of Labor:				**0.001**
No Labor	89 (56.3%)	46 (64.8%)	43 (49.4%)	0.076
Spontaneous	22 (13.9%)	15 (21.1%)	7 (8.0%)	**0.033**
Spontaneous Augmented	9 (5.7%)	2 (2.8%)	7 (8.0%)	0.287
Indication for Delivery^#^:				**<0.001**
Indicated Prelabor Caesarean	3 (1.9%)	3 (4.2%)	0 (0.0%)	0.177
Placental Abnormality	4 (2.5%)	4 (5.6%)	0 (0.0%)	0.083
Fetal Indication	7 (4.4%)	6 (8.5%)	1 (1.1%)	0.067
Other Medically Indicated	10 (6.3%)	1 (1.4%)	9 (10.3%)	**0.049**
Infant Gender, male	83 (52.5%)	41 (57.7%)	42 (48.3%)	0.305
Gestational Age, weeks	37.25 ± 2.89	34.72 ± 2.11	39.32 ± 1.39	**<0.001**
Birth Weight, grams	2999.32 ± 727.20	2499.87 ± 674.09	3406.91 ± 470.70	**<0.001**
Birth Weight Percentile	54.84 ± 28.38	50.93 ± 25.67	58.03 ± 30.19	0.129
SGA	6 (3.8%)	2 (2.8%)	4 (4.6%)	0.555
LGA	28 (17.7%)	9(12.7%)	19(21.8%)	0.197
APGAR at 1 Minute	7.64 ± 1.79	7.34 ± 1.93	7.89 ± 1.63	**0.040**
APGAR at 5 Minutes	8.63 ± 1.09	8.45 ± 1.50	8.77 ± 0.54	0.616

Summary of relevant maternal demographics, clinical characteristics, obstetric outcomes, and neonatal parameters across delivery term classifications: Term (≥ 37 weeks) and Preterm (< 37 weeks).Continuous variables are presented as mean ± standard deviation (SD), with comparisons assessed using the Kruskal-Wallis test. Categorical variables are displayed as counts (n) and percentages (%), with comparisons evaluated using the Chi-Square test. Statistically significant differences are denoted in bold (*p* < 0.05). Abbreviations: PPROM = Preterm premature rupture of the membranes, SGA = Small for gestational age, LGA = Large for gestational age, APGAR = Scoring for appearance, pulse, grimace, activity, and respiration.

# Note: All deliveries in this study were Cesarean.

**Table 2:** Comparisons of MNP concentrations in placentae across the overall cohort, preterm, and term groups.

Micro- and Nanoplastics (MNPs) in μg/g placental tissue	Overall Cohort (*N* = 158)	Preterm Group (*n* = 71)	Term Group (*n* = 87)	*p*-value
Polyethylene (PE)	102.6 ± 117.6	105.3 ± 135.0	88.11 ± 96.12	0.44
Styrene-butadiene rubber (SBR)	33.85 ± 29.18	28.49 ± 30.33	34.99 ± 28.34	0.17
Polyvinyl chloride (PVC)	15.18 ± 12.49	15.54 ± 14.44	13.27 ± 10.70	**0.045**
Polypropylene (PP)	13.63 ± 12.49	14.63 ± 14.36	11.78 ± 9.87	0.087
Nylon 66 (N66)	10.06 ± 8.41	9.03 ± 8.35	10.15 ± 8.69	0.43
Polyethylene terephthalate (PET)	8.62 ± 10.77	11.68 ± 12.66	5.49 ± 8.18	**<0.001**
Nylon 6 (N6)	6.22 ± 15.05	7.48 ± 20.72	4.72 ± 7.55	0.22
Polymethyl methacrylate (PMMA)	2.81 ±2.81	2.77 ± 3.28	2.63 ± 2.36	0.35
Acrylonitrile butadiene styrene (ABS)	1.73 ± 3.83	1.35 ± 3.68	1.92 ± 3.93	**0.02**
Polyurethane (PU)	2.63 ± 4.49	3.86 ± 5.42	1.50 ± 3.27	**<0.001**
Polycarbonate (PC)	1.20 ± 1.74	1.39 ± 2.54	0.95 ± 0.51	**0.007**
Polystyrene (PS)	0.534 ± 3.25	0.534 ± 3.25	0.04 ± 0.22	0.11
Cumulative	197.62 ± 159.91	224.67 ± 180.69	175.54 ± 137.88	**0.038** ^#^

Values are expressed as mean ± SD. *P*-values were calculated using Wilcoxon tests to compare preterm and term groups, with statistical significance indicated in bold (*p* < 0.05).

# Cumulative MNPs were analyzed using log1p-normalized data in the Wilcoxon tests.

## Data Availability

Raw MNP concentrations and one-hot encoded clinical data are available in Table S1a. Custom scripts have been deposited and are publicly available: https://github.com/MADscientist314/Elevated-Micro-and-Nanoplastics-Detected-in-Preterm-Human-Placentae.
